# Nutritional Management of Liver Failure in the Intensive Care Unit

**DOI:** 10.3390/medicina61071210

**Published:** 2025-07-03

**Authors:** Zsófia Verzár, Rudolf Kiss, Csaba Pál Bálint, Annamária Pakai, Tímea Csákvári

**Affiliations:** 1 Institute of Emergency Care, Pedagogy of Health and Nursing Sciences, Faculty of Health Sciences, University of Pécs, H-7621 Pécs, Hungary; 2 Heart Institute, Medical School, University of Pécs, H-7624 Pécs, Hungary; 3 Doctoral School of Health Sciences, Faculty of Health Sciences, University of Pécs, H-7621 Pécs, Hungary; 4 Department of Health Economics and Health Care Management, Institute of Health Insurance, Faculty of Health Sciences, University of Pécs, H-8900 Zalaegerszeg, Hungary

**Keywords:** acute liver failure, chronic liver failure, nutritional management, intensive care

## Abstract

Liver failure, both acute and chronic, represents a complex, life-threatening condition frequently requiring intensive care unit (ICU) admission. Nutritional management is a crucial component of supportive therapy, aiming to mitigate catabolism, preserve lean body mass, and support immune and organ function. In acute liver failure (ALF), early nutritional intervention within 24–48 h and individualized energy–protein prescriptions are essential, even in the presence of hepatic encephalopathy. Chronic liver failure (CLF) and acute-on-chronic liver failure (ACLF) are often associated with severe malnutrition, sarcopenia, and systemic inflammation, necessitating tailored nutritional strategies. Subjective Global Assessment (SGA) and Royal Free Hospital Global Assessment (RFH-GA) tools are instrumental in identifying nutritional risk. Enteral nutrition (EN) is preferred across all stages, with parenteral nutrition (PN) reserved for contraindications. Special considerations include micronutrient repletion, prevention of refeeding syndrome, and perioperative nutritional support in transplant candidates and recipients. This clinical overview summarizes current evidence and guidelines on ICU nutrition in liver failure, emphasizing a multidisciplinary approach to improve outcomes.

## 1. Introduction

Malnutrition is a highly prevalent and prognostically significant complication in patients with chronic liver disease, particularly those with cirrhosis and acute-on-chronic liver failure (ACLF). These patients frequently experience profound metabolic disturbances, including sarcopenia, micronutrient deficiencies, and altered energy and protein requirements.

Several major international societies have published guidelines addressing nutrition in liver failure, reflecting the importance of nutritional management in these patients. The European Society for Clinical Nutrition and Metabolism (ESPEN) provides detailed recommendations on nutritional support in both acute and chronic liver disease, emphasizing individualized care [1]. Similarly, the European Association for the Study of the Liver (EASL) includes nutrition as a key component of its clinical practice guidelines for managing liver conditions, particularly cirrhosis [2]. In the United States, the American Association for the Study of Liver Diseases (AASLD) acknowledges the role of nutrition in the management of hepatic disorders, especially in preventing and treating malnutrition and sarcopenia [3]. The American College of Gastroenterology (ACG) also offers guidance that incorporates nutritional considerations, particularly in the context of complications such as hepatic encephalopathy [4]. Together, these organizations underscore the consensus on the critical role of nutrition in improving outcomes for patients with liver failure.

In the intensive care setting, nutritional management becomes even more complex due to systemic inflammation, organ dysfunction, and the risk of refeeding syndrome. Furthermore, for candidates awaiting liver transplantation (LT), nutritional status is directly linked to waitlist mortality, postoperative complications, and overall outcomes. Despite growing recognition of its clinical importance, nutritional therapy in liver failure remains inadequately implemented in practice. This gap is not due to a lack of evidence—recent guidelines from AASLD, EASL, and ACG provide clear recommendations on energy and protein targets—but rather reflects a combination of factors: limited awareness among non-nutrition specialists, variability in institutional protocols, and logistical challenges in delivering timely enteral support, particularly in critically ill patients. As a result, malnutrition, sarcopenia, and frailty continue to be prevalent and under-treated contributors to poor outcomes in both acute and chronic liver failure settings. This review aims to provide a comprehensive, evidence-based overview of nutritional assessment and support strategies in patients with chronic liver failure and ACLF, with a particular focus on those in the intensive care unit and those awaiting liver transplantation.

## 2. Acute Liver Failure (ALF)

### 2.1. Definition and Overview, Etiology

Acute liver failure (ALF) is defined as the rapid development of hepatic dysfunction, manifesting with jaundice, coagulopathy (INR ≥ 1.5), and hepatic encephalopathy, in patients without prior cirrhosis or known liver disease. This syndrome typically evolves within 26 weeks of the initial liver insult and is associated with high short-term mortality [5]. The hallmark of ALF is the sudden loss of hepatocellular function, which may lead to multi-organ failure, including renal, respiratory, and cardiovascular dysfunction. Immediate ICU-level care is essential for hemodynamic support, airway protection, and neurological monitoring [6].

**Figure 1:**
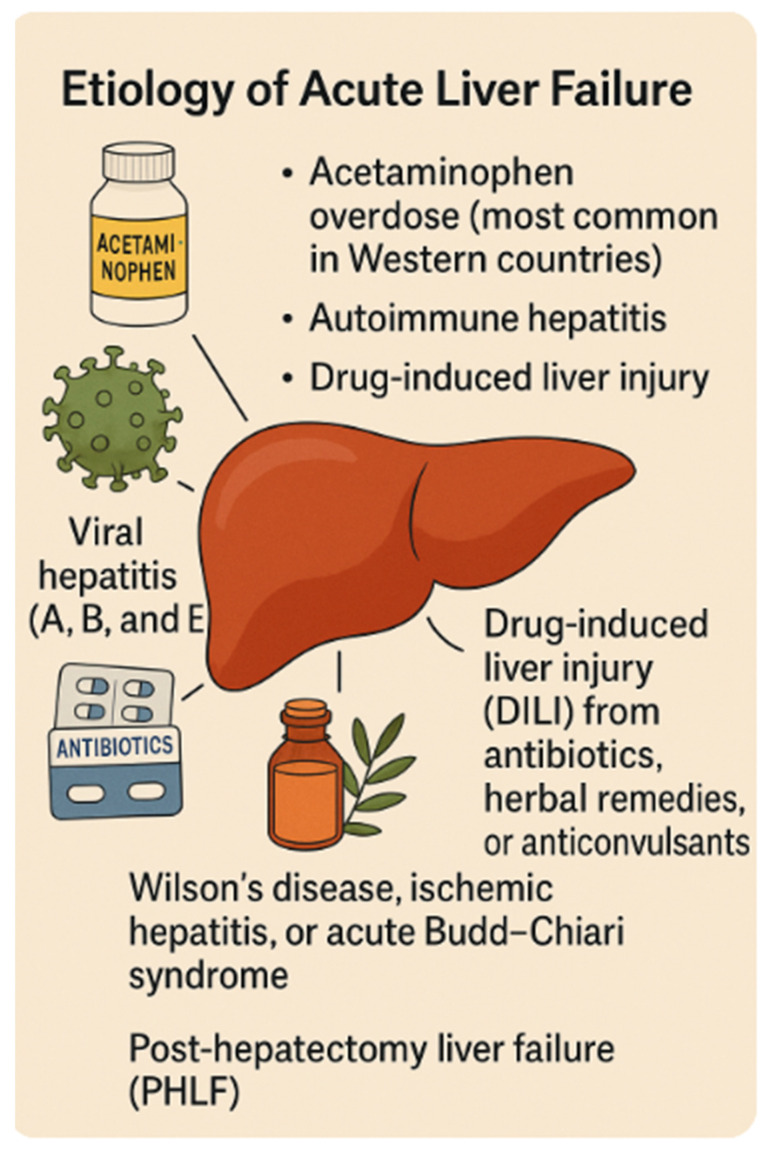
Etiology of acute liver failure, including viral hepatitis, drug-induced liver injury, autoimmune causes, and iatrogenic factors (e.g., post-hepatectomy liver failure).

The most common causes of acute liver failure (ALF) vary by geographic region. In Western countries, acetaminophen overdose represents the leading etiology. Other frequent causes include viral hepatitis—specifically types A, B, and E—autoimmune hepatitis, and drug-induced liver injury (DILI), which may result from exposure to antibiotics, herbal remedies, or anticonvulsants. Less commonly, ALF may be attributed to Wilson’s disease, ischemic hepatitis, acute Budd–Chiari syndrome, or post-hepatectomy liver failure (PHLF) [7]. Notably, in up to 20% of cases, the underlying cause remains indeterminate [8] (Figure 1).

### 2.2. Clinical Presentation and Diagnosis

Patients with acute liver failure (ALF) typically present with a rapid onset of jaundice, altered mental status indicative of hepatic encephalopathy, and coagulopathy. Additional clinical features often include renal dysfunction, lactic acidosis, and hypoglycemia. The diagnostic workup comprises laboratory testing—such as aspartate aminotransferase (AST), alanine aminotransferase (ALT), bilirubin, international normalized ratio (INR), and ammonia levels—along with liver imaging to rule out obstructive or vascular etiologies. Serologic and toxicology screens are also essential components of the diagnostic process.

Prognostic evaluation frequently relies on the King’s College Criteria, the Model for End-Stage Liver Disease (MELD) score, and dynamic indicators such as lactate and ammonia concentrations [9]. Among these, the King’s College Criteria is a widely adopted prognostic tool used to identify patients who are unlikely to survive without emergency liver transplantation. These criteria are stratified based on the etiology of ALF, with distinct parameters for acetaminophen-induced and non-acetaminophen-related cases.

In acetaminophen-induced ALF, transplantation is indicated if the arterial pH is less than 7.30 following adequate fluid resuscitation, or if all three of the following criteria are met: INR greater than 6.5, serum creatinine exceeding 300 μmol/L (3.3 mg/dL), and the presence of grade III or IV hepatic encephalopathy.

For non-acetaminophen ALF, transplantation is recommended if the INR exceeds 6.5, or if three or more of the following factors are present: age younger than 10 or older than 40 years; etiology involving non-A, non-B hepatitis, halothane exposure, or idiosyncratic drug reactions; duration of jaundice longer than seven days prior to the onset of encephalopathy; INR greater than 3.5; and serum bilirubin levels exceeding 300 μmol/L (17.5 mg/dL).

In practical use, ALF-specific prognostic systems—such as the King’s College Criteria or the CLIF-C ACLF score—are recommended because the MELD score was originally developed to assess mortality risk in chronic, progressive liver dysfunction [10]. Although MELD incorporates parameters reflecting hepatic and renal function, it does not adequately capture the rapid clinical trajectory or extrahepatic organ failures typical of acute liver failure.

The King’s College Criteria is a decision-focused tool designed to identify patients with acute liver failure who are unlikely to survive without liver transplantation (Table 1). In contrast, the MELD score provides a continuous risk assessment and is primarily used to predict short-term mortality in patients with chronic liver disease. While both tools play a critical role in guiding the timing of referral and listing for liver transplantation, they must be interpreted within the broader clinical context, and sound clinical judgment remains essential.

Management of acute liver failure (ALF) in the intensive care unit (ICU) is centered on supportive therapy, which remains the cornerstone of treatment. Key components include airway protection, particularly in patients with grade III or IV hepatic encephalopathy, as well as hemodynamic stabilization using intravenous fluids and vasopressors when necessary. Continuous monitoring for cerebral edema is essential, with prompt intervention if detected. Metabolic disturbances such as hypoglycemia, acidosis, and coagulopathy must be corrected proactively. In cases where sepsis is suspected, empirical antibiotic therapy should be initiated without delay.

N-Acetylcysteine (NAC) is indicated not only in acetaminophen toxicity but may also improve transplant-free survival in non-acetaminophen ALF [11].

Orthotopic liver transplantation (OLT) remains the definitive treatment for patients with acute liver failure (ALF) who are unlikely to recover spontaneously. As outcomes are highly time-sensitive, early initiation of transplant evaluation is essential for all patients presenting with severe ALF [12].

Prognosis in ALF is highly variable, with mortality rates ranging from 20% to 80%, depending on the underlying etiology and the presence of complications. Spontaneous recovery is most commonly observed in cases of acetaminophen-induced ALF, whereas patients with indeterminate or drug-induced etiologies typically have poorer outcomes in the absence of transplantation [5].

## 3. Nutrition Therapy in Acute Liver Failure (ICU Setting)

Patients with acute liver failure (ALF) commonly present with a hypermetabolic and catabolic state, characterized by increased protein degradation, disrupted glucose homeostasis, and impaired nutrient metabolism. These metabolic derangements are often intensified by hepatic encephalopathy (HE), systemic inflammation, and multi-organ dysfunction, all of which are frequently observed in the intensive care unit (ICU) setting [13,14].

Accurate assessment of energy requirements is essential for optimizing nutritional support [15]. The current gold standard for determining caloric needs is indirect calorimetry (IC), which directly measures oxygen consumption (VO_2_) and carbon dioxide production (VCO_2_) to calculate resting energy expenditure (REE). IC is especially valuable in liver failure, where metabolic rates may vary significantly based on encephalopathy grade, infection, and organ support therapies (e.g., CRRT, vasopressors).

When indirect calorimetry is not available, modern ICU-specific predictive equations are preferred over generic formulas. Among these, the Penn State equation (2003b or 2010 variant) has shown better accuracy in ventilated patients, particularly when body temperature and minute ventilation are factored in. However, even these models have wide margins of error, and clinicians should interpret results with caution and reassess regularly based on clinical trajectory.

For practical use, a range of 25–30 kcal/kg/day is typically recommended, with adjustments based on tolerance, metabolic demand, and organ dysfunction. Daily re-evaluation is essential in dynamic ICU conditions. Table 2 summarizes recommendations tailored to the ICU setting [5,11].

Unlike in chronic hepatic encephalopathy, the role of branched-chain amino acids (BCAAs) in acute hepatic encephalopathy due to ALF is unestablished. The available data consist of small case series and animal models, with no robust randomized trials demonstrating benefit in this setting. Clinicians should therefore consider BCAA use in ALF only on a case-by-case basis, when standard nutritional strategies are insufficient, and within research or highly monitored protocols.

### 3.1. Enteral vs. Parenteral Nutrition

Enteral nutrition (EN) is the preferred route of nutritional support in patients with acute liver failure, provided the gastrointestinal tract remains functional. EN offers several advantages, including a reduced risk of infection, preservation of gut mucosal integrity, and potential modulation of the immune response. When EN is contraindicated or fails to meet nutritional needs—such as in cases of ileus or gastrointestinal bleeding—parenteral nutrition (PN) may be initiated. In these situations, low-glucose, lipid-sparing formulations are recommended, with close monitoring of triglyceride, glucose, and ammonia levels to minimize complications. Early nutritional intervention is essential and should ideally begin within 24 to 48 h of ICU admission [16].

### 3.2. Micronutrients and Vitamins

Patients with acute liver failure (ALF) frequently present with significant micronutrient deficiencies, which require routine supplementation. Thiamine is essential to prevent Wernicke’s encephalopathy, while zinc supports urea cycle function and facilitates ammonia detoxification. Selenium and magnesium play critical roles in maintaining mitochondrial function. Additionally, absorption of fat-soluble vitamins (A, D, E, and K) is often impaired due to reduced bile production, necessitating proactive supplementation. Electrolyte disturbances, particularly hypophosphatemia and hyponatremia, should be corrected promptly to prevent further complications.

Ongoing monitoring and adjustment of nutritional therapy are vital in the ICU setting. Daily evaluation of nutritional status is recommended and should include serum markers such as albumin and prealbumin (tracked over time), nitrogen balance when feasible, and clinical indicators such as weight, fluid balance, and the need for organ support. Close collaboration with clinical nutrition specialists is strongly advised for optimal management of ALF. Individualized nutrition strategies can significantly improve outcomes in critically ill patients with liver failure by reducing complications, shortening ICU length of stay, and enhancing survival rates [14].

While current guidelines offer general recommendations, their application must be adapted to the specific demands of the ICU environment. The integration of trained nutrition experts into multidisciplinary care teams should be considered a standard component of high-quality critical care for ALF patients.

## 4. Nutrition in Critically Ill Patients with Cirrhosis: Route, Impact, and Clinical Considerations

Malnutrition and altered metabolic response are nearly universal in critically ill patients with chronic liver failure (CLF), and they significantly worsen outcomes. In the intensive care setting, nutritional support becomes a core therapeutic strategy—not merely supportive care. Poor nutritional status in this population is associated with increased risk of hepatic encephalopathy, infections, impaired wound healing, muscle wasting, prolonged mechanical ventilation, and increased ICU mortality.

### 4.1. Impact of Nutrition on Outcomes and Complications

Critical illness in cirrhosis is marked by hypercatabolism, insulin resistance, and accelerated muscle proteolysis. Adequate nutritional therapy in the ICU helps to preserve lean body mass and prevent sarcopenia, which strongly correlates with mortality and ventilator dependency; reduce the risk and severity of hepatic encephalopathy by providing consistent energy and protein intake, stabilizing ammonia metabolism; support immune function, thereby lowering the risk of spontaneous bacterial peritonitis, sepsis, and nosocomial infections; and also improve nitrogen balance, hepatic synthetic capacity, and facilitate hepatic regeneration.

In cirrhosis, particularly in critically ill patients, maintaining adequate protein intake is also essential to prevent muscle wasting, hepatic encephalopathy (HE), and immune dysfunction. The type of protein—animal vs. vegetable (plant-based)—can influence metabolic outcomes, ammonia production, and tolerance, especially in patients prone to HE (Table 3).

**Table 3:** Evidence-based comparison of protein sources in cirrhosis management.

Protein Source	Advantages	Concerns	Supporting Evidence
Animal protein	High biological value, complete amino acid profile	Higher ammonia generation, less tolerated in HE	[17]
Vegetable protein	Lower ammonia production, higher fiber content	Lower biological value (balanced with variety)	[18]
BCAA supplements	Reduces HE recurrence, supports muscle metabolism	Cost, compliance	[19,20]
Mixed protein diet	Flexibility, adequate intake	Depends on individual tolerance	General ICU practice

Abbreviations: BCAAs = branched-chain amino acids; HE = hepatic encephalopathy.

1. Vegetable proteins are often better tolerated in patients with cirrhosis, particularly in those with hepatic encephalopathy. This is due to several factors, including a lower production of ammonia and aromatic amino acids (AAAs), as well as a higher dietary fiber content, which enhances nitrogen excretion through the gastrointestinal tract. In addition, vegetable proteins exert a favorable effect on the gut microbiota and support improved colonic transit.
2. A study by Bianchi et al. [18] showed that cirrhotic patients receiving vegetable protein diets had fewer episodes of hepatic encephalopathy and better nitrogen balance than those on animal protein diets at the same protein dose.
3. BCAAs (branched-chain amino acids)—found more abundantly in vegetable and dairy proteins or given as supplements—have been shown in multiple trials and meta-analyses (e.g., [19]) to improve outcomes in HE and reduce muscle loss.

Branched-chain amino acids (BCAAs) have demonstrated clinical benefits in cirrhosis, particularly in hepatic encephalopathy and sarcopenia. Multiple randomized controlled trials and meta-analyses report improved mental status, nitrogen balance, and reduced HE recurrence with BCAA supplementation [19,20,21]. In critically ill patients, BCAA-enriched enteral formulas may support metabolic stability and improve feeding tolerance.

4. Despite the traditional caution, animal proteins are not contraindicated, and restriction is not necessary. The key is individual tolerance. Many patients can tolerate mixed diets if encephalopathy is controlled.

### 4.2. Clinical Implications in the ICU Setting

In patients with overt or recurrent hepatic encephalopathy, vegetable-based proteins or branched-chain amino acid (BCAA)-enriched formulas may be preferred. Mixed protein diets that combine animal and plant sources are also acceptable, provided they are well tolerated. In the context of critical illness, ensuring adequate protein intake—typically 1.2 to 1.5 g/kg/day—is more important than adhering strictly to specific protein sources. Additionally, the use of elemental or semi-elemental enteral formulas enriched with BCAAs in the intensive care setting may help optimize nitrogen balance while minimizing ammonia production.

While both animal and plant-based proteins can be used in cirrhosis, plant-based proteins and BCAA-enriched formulas may offer superior tolerability, especially in patients with hepatic encephalopathy. The choice should be individualized, and evidence supports the safety and efficacy of high-protein diets from mixed sources when guided by clinical response rather than blanket restriction [17,18,19].

### 4.3. Preferred Route of Nutrition in the ICU Setting

Studies have shown that early and individualized nutritional intervention in ICU cirrhotic patients is associated with shorter ICU stay and reduced complications, especially when initiated within the first 24–48 h of admission.

Enteral nutrition (EN) is the first-line approach even in the presence of liver dysfunction, provided there are no contraindications such as uncontrolled GI bleeding or ileus. EN maintains gut mucosal integrity, reduces bacterial translocation, and supports immune function.

When EN is not feasible or fails to meet targets, parenteral nutrition (PN) becomes necessary. In such cases, special care must be taken to start PN slowly to prevent refeeding syndrome, particularly in severely malnourished patients; serum phosphate, magnesium, and glucose should be monitored closely; and lipid emulsions should be adjusted based on hepatic clearance capacity and monitored for hypertriglyceridemia.

### 4.4. Optimum Feeding Strategy in the ICU

In patients with chronic liver failure (CLF) admitted to the intensive care unit (ICU), nutritional management should aim to provide 25–30 kcal/kg/day, adjusted according to the patient’s actual body weight. Protein intake should range between 1.2 and 1.5 g/kg/day, and protein restriction is not recommended even in cases of hepatic encephalopathy (HE). Continuous enteral feeding is preferred; however, if full feeding is not tolerated, trophic feeding may be considered. Nutritional support should be initiated within 24 to 48 h, provided the patient is hemodynamically stable. A balanced macronutrient composition is recommended, with individual adjustments to carbohydrate and fat intake based on the patient’s tolerance. Attention should also be given to micronutrient supplementation, particularly zinc, thiamine, selenium, phosphate, magnesium, and fat-soluble vitamins (A, D, E, and K). Ongoing monitoring should include daily assessments of gastrointestinal tolerance, ammonia levels, fluid balance, and infection markers.

To mitigate fasting-induced catabolism, the use of a late evening snack (LES)—typically a 50 g complex carbohydrate meal—has demonstrated benefits in cirrhosis, including improved nitrogen balance, lean body mass, and reduced ammonia levels [22,23]. In the ICU setting, while a true LES may not be feasible, the strategy is mimicked by continuous or evenly spaced enteral nutrition to limit catabolic phases.

### 4.5. Micronutrient Supplementation

Due to impaired absorption and increased metabolic demands, patients with chronic liver failure (CLF) in the intensive care unit often require targeted micronutrient supplementation. Zinc is essential for ammonia detoxification and wound healing, while thiamine supplementation is particularly important in cases of alcohol-related liver disease. Additional supplementation with selenium, magnesium, and phosphate may also be necessary. Furthermore, fat-soluble vitamins (A, D, E, and K) should be closely monitored and replaced as needed, especially in patients with cholestatic liver disease. These micronutrients form an integral part of the broader metabolic management in this patient population.

### 4.6. Dynamic Monitoring and Individualization

Nutritional therapy should be dynamically individualized, with careful consideration of several clinical factors, including the patient’s ventilatory status, the grade of hepatic encephalopathy, gastrointestinal function, fluid and electrolyte balance, as well as renal and hepatic support modalities such as continuous renal replacement therapy (CRRT) and vasopressor use. Safe and effective nutritional management in this complex setting requires the coordinated effort of a multidisciplinary intensive care unit team, including intensivists, hepatologists, pharmacists, and clinical nutritionists.

Nutritional interventions in cirrhosis have been shown to significantly impact clinical outcomes, particularly in critically ill patients. Table 4 summarizes key strategies and the supporting evidence. Late evening snacks (LESs) improve nitrogen balance and reduce overnight catabolism, while branched-chain amino acid (BCAA) supplementation improves encephalopathy and muscle mass preservation. Plant-based proteins are better tolerated in patients with hepatic encephalopathy due to lower ammonia generation and higher fiber content. Adequate protein intake, regardless of source, is critical to prevent sarcopenia and immune dysfunction. Enteral nutrition remains the preferred route of feeding, and targeted micronutrient supplementation further supports metabolic and neurologic stability.

## 5. Chronic Liver Failure (CLF)

### 5.1. Definition and Pathophysiology

Chronic liver failure (CLF) represents the terminal stage of chronic liver disease, typically resulting from progressive hepatic fibrosis and cirrhosis. It is marked by irreversible hepatocellular dysfunction, the development of portal hypertension, and clinical signs of hepatic decompensation such as ascites, variceal bleeding, and hepatic encephalopathy [25]. The condition arises from a variety of underlying etiologies—including viral hepatitis, alcohol-related liver disease, nonalcoholic steatohepatitis (NASH), autoimmune liver diseases, and metabolic disorders—and progresses over several years, often without overt symptoms until decompensation manifests.

### 5.2. Clinical Features and Complications

**Figure 2:**
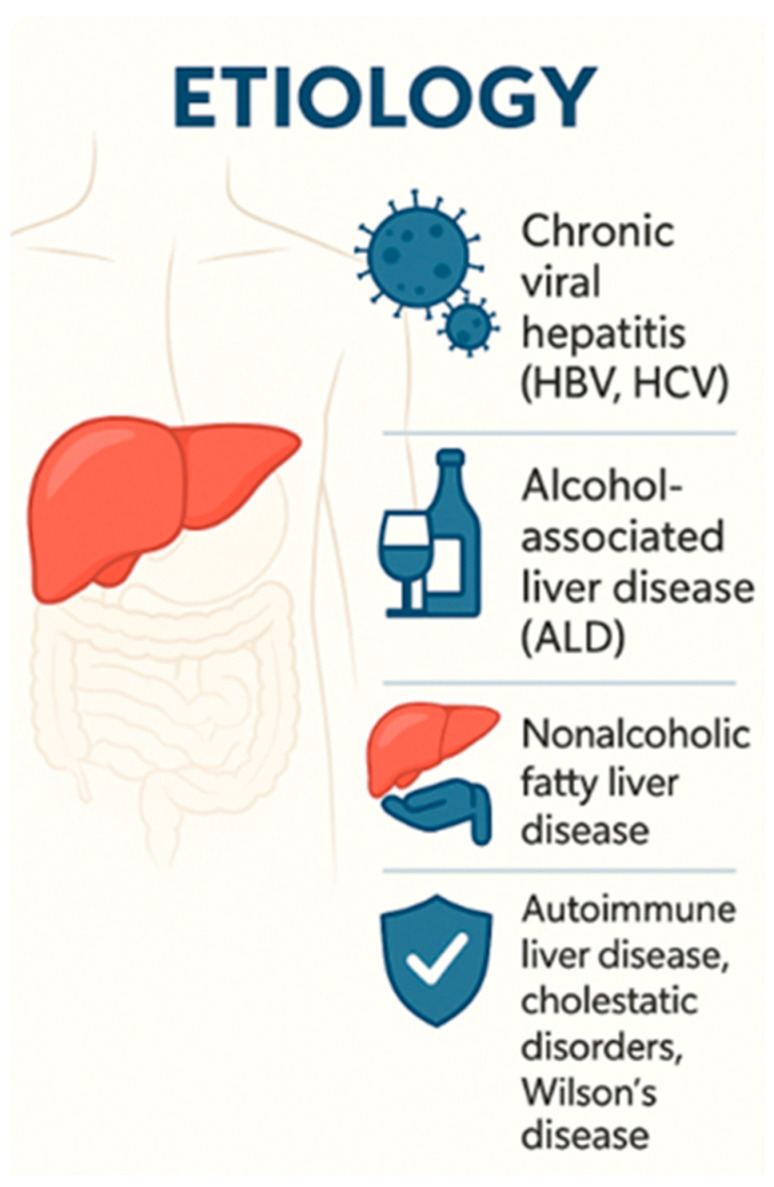
Etiology of chronic liver failure, including viral hepatitis, alcohol-related liver disease, NASH, and autoimmune and metabolic liver diseases.

The hallmark clinical manifestations of CLF and cirrhosis decompensation include ascites, jaundice, variceal hemorrhage, hepatic encephalopathy (HE), spontaneous bacterial peritonitis (SBP), and hepatorenal syndrome (HRS). Decompensated cirrhosis is also associated with significant immune dysfunction, which increases vulnerability to infections and sepsis. In fact, infections are among the most common causes of hospitalization and intensive care unit (ICU) admission in this patient population [26] (Figure 2).

Patients with chronic liver failure (CLF) may require intensive care unit (ICU) admission due to severe complications such as massive gastrointestinal bleeding, grade III–IV hepatic encephalopathy, acute kidney injury or hepatorenal syndrome, sepsis or septic shock, refractory ascites requiring large-volume paracentesis, and acute-on-chronic liver failure (ACLF). These conditions are associated with markedly increased morbidity and mortality and necessitate intensive organ support, strict fluid balance management, and advanced hemodynamic and neurological monitoring.

### 5.3. Liver Transplantation Consideration

Timely evaluation for liver transplantation is essential for patients with advanced CLF. Early referral improves outcomes, particularly as many of these patients may deteriorate rapidly. Prognostic scoring systems, including the Model for End-Stage Liver Disease (MELD) score and the Child–Pugh classification, provide guidance in assessing transplant eligibility and predicting post-transplant outcomes [27].

Moreover, recent findings suggest that liver fibrosis indices, such as the FIB-4 score, may be associated with worse outcomes in critically ill patients with COVID-19, supporting their potential role in risk stratification beyond chronic liver disease alone [28].

Non-invasive fibrosis scores, such as the FIB-4, NAFLD fibrosis score, and BARD score, have been validated for the assessment of advanced fibrosis in NAFLD patients, and may assist in stratifying nutritional and transplant-related risk in the ICU [29].

### 5.4. Nutrition Therapy in Chronic Liver Failure (ICU Setting)

Malnutrition is a highly prevalent and clinically significant complication in CLF, affecting up to 80% of patients with cirrhosis. It is frequently underrecognized due to confounding factors such as ascites and peripheral edema, which mask true weight loss and lean body mass reduction [13]. Malnutrition in this population is associated with an increased risk of hepatic encephalopathy, impaired immune function, delayed wound healing, and higher ICU mortality.

### 5.5. Nutritional Assessment

Accurate nutritional assessment in patients with chronic liver failure (CLF) is frequently complicated by clinical features such as ascites and peripheral edema, which obscure true weight changes and body composition. One of the most widely utilized tools in this setting is the Subjective Global Assessment (SGA), a validated clinical method that stratifies patients into three categories of nutritional status: SGA-A (well-nourished), SGA-B (moderate or suspected malnutrition), and SGA-C (severe malnutrition). The SGA incorporates multiple domains, including recent unintentional weight loss, decreased dietary intake, gastrointestinal symptoms, functional capacity, and physical examination findings such as loss of subcutaneous fat and muscle wasting. Its ease of use, non-reliance on laboratory parameters, and reproducibility make it especially advantageous in ICU environments, where rapid and reliable bedside assessments are essential for guiding nutritional interventions.

A 2025 study by Miwa et al. demonstrated that SGA effectively identifies energy malnutrition and correlates with increased mortality risk in patients with liver cirrhosis. Patients classified as SGA-B or SGA-C showed significantly higher risk of poor outcomes [30].

Complementary tools such as mid-arm muscle circumference, handgrip strength, or CT-based sarcopenia assessment can further enhance the evaluation, especially in patients with fluid retention.

Another validated tool is the Royal Free Hospital Global Assessment (RFH-GA), which combines subjective and objective parameters, including body mass index (BMI), mid-arm muscle circumference (MAMC), and dietary intake, to assess nutritional status in patients with liver cirrhosis. The RFH-GA has been shown to be effective in identifying malnutrition and predicting clinical outcomes in this patient population [31].

Accurate nutritional assessment in patients with decompensated chronic liver failure remains challenging due to confounding factors such as fluid retention and inflammation. Validated tools such as the Subjective Global Assessment (SGA) and the Royal Free Hospital Global Assessment (RFH-GA) are commonly employed to evaluate nutritional status. In addition to clinical scoring systems, objective measures—including mid-arm muscle circumference, handgrip strength, and computed tomography (CT)-based assessment of sarcopenia—can provide valuable insights into muscle mass and function. Traditional biochemical markers such as serum albumin and prealbumin, however, are considered unreliable in this population, as their levels are often influenced by impaired hepatic synthetic function and systemic inflammation rather than nutritional status alone.

### 5.6. Energy and Protein Requirements

Patients with chronic liver failure (CLF) typically exhibit a hypermetabolic state characterized by accelerated skeletal muscle catabolism and reduced glycogen storage. As a result, energy and protein requirements are elevated, with recommended targets of 30–35 kcal/kg/day and 1.2–1.5 g/kg/day of protein, respectively. To mitigate fasting-induced catabolism, frequent small meals are advised, including a late evening carbohydrate-rich snack. Importantly, protein restriction is no longer recommended, even in patients with hepatic encephalopathy, as it can exacerbate malnutrition and worsen outcomes. In cases of refractory or uncontrolled hepatic encephalopathy, however, the use of branched-chain amino acid (BCAA)-enriched formulas may be beneficial [13,32].

### 5.7. Route of Nutrition

Enteral nutrition (EN) is the preferred route of nutritional support in critically ill patients with chronic liver failure (CLF), as it helps preserve gut barrier integrity, reduces the risk of bacterial translocation and sepsis, and supports intestinal immune function. When enteral nutrition is contraindicated or fails to meet nutritional requirements, parenteral nutrition (PN) becomes necessary. In such cases, careful attention must be paid to glucose and lipid tolerance, and close monitoring of serum electrolytes—particularly phosphate and magnesium—is essential to prevent refeeding syndrome.

### 5.8. Micronutrient Supplementation

Due to malabsorption and altered nutrient metabolism, patients with chronic liver failure (CLF) often require targeted micronutrient supplementation [33]. Zinc is essential for ammonia detoxification and immune function, while thiamine supplementation is critical, particularly in individuals with alcohol-related liver disease or a high risk of encephalopathy. Supplementation of fat-soluble vitamins (A, D, E, and K), as well as selenium, magnesium, and phosphate, is also necessary, especially during refeeding periods.

Ongoing clinical monitoring should include daily assessment of gastrointestinal tolerance, nitrogen balance, and organ function. Particular attention must be paid to the progression of hepatic encephalopathy, fluid status, and signs of infection. Nutritional therapy should be dynamically adjusted based on disease progression and the patient’s need for organ support. Effective management requires a multidisciplinary approach involving hepatologists, intensivists, and clinical nutrition specialists to optimize outcomes.

## 6. Acute-on-Chronic Liver Failure (ACLF)

Acute-on-chronic liver failure (ACLF) is characterized by an acute decompensation of chronic liver disease, associated with the development of one or more organ failures and a high short-term mortality risk. It typically occurs in the context of systemic inflammation and identifiable precipitating events [34]. In contrast to classical decompensated cirrhosis, ACLF presents with a rapid decline in organ function and displays distinct immunological and hemodynamic alterations.

According to the EASL-CLIF Consortium, diagnostic criteria for ACLF include the presence of underlying chronic liver disease (most commonly cirrhosis), an acute precipitating event, and the development of organ failure(s) as defined by the CLIF-SOFA score, which assesses liver, kidney, brain, coagulation, circulatory, and respiratory function. The 28-day mortality rate increases proportionally with the number of organ failures.

Common precipitating factors include spontaneous bacterial peritonitis (SBP), pneumonia, bloodstream infections, acute alcoholic hepatitis, gastrointestinal bleeding, drug-induced liver injury, and acute viral hepatitis superimposed on cirrhosis.

In some cases, no clear trigger is found, especially in patients with already advanced portal hypertension and systemic inflammation.

EASL and AASLD align on an evidence-based approach to ACLF: early aggressive nutrition with high protein and adequate energy intake, avoidance of fasting, methodical micronutrient correction, and organ-failure-based prognostication using structured scoring. Feeding strategies must be seamlessly integrated into critical care workflows to optimize patient recovery (Table 5).

### 6.1. Immunopathology and Systemic Inflammation

ACLF is marked by profound immune dysregulation, which plays a central role in the pathogenesis of organ failure. This state is characterized by overactivation of innate immune cells and a cytokine storm-like profile, including elevated levels of interleukin-6 (IL-6), tumor necrosis factor-alpha (TNF-α), and other pro-inflammatory mediators. Additionally, mitochondrial dysfunction and increased oxidative stress further exacerbate systemic injury. This exaggerated inflammatory response directly contributes to the development of organ failures, particularly acute kidney injury and respiratory dysfunction.

### 6.2. ICU Admission and Management

ACLF is among the most common indications for ICU admission in patients with cirrhosis. Effective management requires a comprehensive, multisystem approach that includes hemodynamic stabilization with fluids and vasopressors, ventilatory support in cases of acute respiratory distress syndrome (ARDS) or grade III–IV hepatic encephalopathy, and renal replacement therapy (RRT) for acute kidney injury (AKI) or hepatorenal syndrome (HRS). Prompt administration of antibiotics and source control are essential for managing infections, alongside strict measures to prevent nosocomial infections. Prognostic tools such as the CLIF-C ACLF and MELD scores are instrumental in guiding both clinical decision-making and transplantation eligibility.

### 6.3. Prognosis and Transplant Candidacy

Mortality in ACLF increases in direct proportion to the number of failing organs, with ACLF grade 3—defined by failure of three or more organs—associated with a 28-day mortality exceeding 80%. Urgent liver transplantation remains the only definitive treatment in such cases. Therefore, early recognition and expedited transplant evaluation are critical to improving outcomes.

## 7. Nutritional Therapy in ACLF: Special Considerations in Multi-Organ Failure

Acute-on-chronic liver failure (ACLF) is characterized by systemic inflammation, rapidly evolving organ dysfunction, and exceptionally high metabolic demands. Nutritional therapy in this setting must address the interplay between catabolism, immune dysfunction, and the impact of life-sustaining interventions such as mechanical ventilation, vasopressors, and continuous renal replacement therapy (CRRT).

Patients with acute-on-chronic liver failure (ACLF) face a number of unique nutritional and metabolic challenges. One of the key issues is a markedly increased catabolic state, which leads to rapid muscle wasting and proteolysis that can occur within just a few days of admission to the intensive care unit. Additionally, gastrointestinal function is often impaired in these patients, significantly limiting the feasibility of enteral nutrition in many cases. There is also a high risk of refeeding syndrome, particularly among individuals with alcohol-related cirrhosis, who frequently present with profound phosphate and magnesium depletion. Furthermore, increased losses of micronutrients are commonly observed due to continuous renal replacement therapy (CRRT) and large-volume paracentesis.

Patients undergoing CRRT experience significant losses of water-soluble nutrients, electrolytes, and amino acids, necessitating specific adaptations in the feeding regimen, as seen in Table 6.

### Clinical Recommendations

Enteral feeding should be initiated within 24 to 48 h, provided the patient is hemodynamically stable. In cases of hepatic encephalopathy or protein intolerance, branched-chain amino acid (BCAA)-enriched formulas may be used selectively. Nitrogen balance should be monitored regularly, and protein targets should be adjusted as needed—up to 2.0 g/kg/day in patients undergoing continuous renal replacement therapy (CRRT). Thiamine supplementation (200–300 mg/day intravenously) and trace elements should be administered routinely during CRRT. Additionally, patients should be closely monitored for signs of refeeding syndrome, particularly during the first 3 to 5 days of nutritional support.

## 8. Nutrition Therapy in Acute-on-Chronic Liver Failure (ACLF)—ICU Perspective

Patients with ACLF experience profound metabolic stress, intense catabolism, and systemic inflammation, all contributing to accelerated muscle wasting and energy deficits [36]. Malnutrition and sarcopenia are not only frequent but also independent predictors of mortality in these patients.

Energy and protein deficits are often underestimated due to fluid overload and hypoalbuminemia. Prompt initiation of nutrition support is essential, ideally within 24–48 h of ICU admission [13].

In patients with ACLF, energy expenditure is often elevated due to systemic inflammation and organ dysfunction. The general target for energy provision is 30–35 kcal/kg/day. When feasible, indirect calorimetry remains the gold standard for assessing energy needs; in its absence, predictive equations adjusted by stress factors ranging from 1.3 to 1.5 are commonly utilized. Ongoing adjustment of caloric intake is essential, taking into account dynamic changes in clinical status, fluid balance, and the level of organ support.

### 8.1. Protein Provision

Despite the presence of hepatic encephalopathy (HE), protein restriction is not advised in patients with ACLF. A daily protein intake of 1.2–1.5 g/kg is recommended, with the potential use of branched-chain amino acid (BCAA)-enriched formulas in those with recurrent HE. Adequate protein is essential for preserving immune function, supporting wound healing, and facilitating ammonia detoxification. Enteral nutrition (EN) is the preferred route whenever feasible, as it helps maintain gut barrier integrity and microbiome stability, reduces bacterial translocation and sepsis risk, and may attenuate systemic inflammation. When EN is contraindicated or insufficient, parenteral nutrition (PN) should be initiated with caution, ensuring careful monitoring to avoid glucose overload and regularly assessing ammonia levels, triglycerides, and lactate.

### 8.2. Micronutrient and Electrolyte Management

ACLF is frequently associated with multiple micronutrient deficiencies. Zinc plays a critical role in modulating ammonia metabolism and supporting immune function, while selenium contributes to antioxidant defense and mitochondrial protection. Magnesium and phosphate are essential, particularly during refeeding, to prevent metabolic complications. Deficiencies in thiamine, vitamin D, and other fat-soluble vitamins (A, E, and K) are also common due to impaired hepatic processing and malabsorption. Micronutrient supplementation should be systematic and guided by laboratory parameters and clinical suspicion.

### 8.3. Monitoring and Adjustments

Daily monitoring is essential in the nutritional management of patients with ACLF. Key parameters include plasma ammonia concentrations, serum electrolytes, and blood glucose levels. Clinicians should also assess for signs of feeding intolerance, such as aspiration, ileus, or high gastric residual volumes. When feasible, nitrogen balance assessments can help guide protein delivery. Nutritional targets should be re-evaluated at least every 48–72 h, with necessary adjustments based on fluid shifts, renal function, and neurological status. Involvement of a clinical nutrition team is essential for outcome improvement and for adapting protocols to the dynamic nature of ACLF in the ICU.

Both AASLD (2024) and ACG (2025) agree that protein restriction is outdated, even in hepatic encephalopathy. Adequate protein intake is essential to prevent sarcopenia and support ammonia detoxification through muscle metabolism [5,11]. In both CLF and ACLF, early nutritional intervention with individualized energy and protein targets, along with proactive micronutrient replacement, is a cornerstone of care. Current guidance from AASLD and ACG highlights the importance of avoiding protein restriction and ensuring routine supplementation of zinc, thiamine, selenium, and fat-soluble vitamins, particularly in critically ill patients (Table 7).

## 9. Nutrition in Liver Transplant Candidates—Importance of Pre-Transplant Nutrition

Malnutrition is a well-established risk factor for adverse surgical outcomes in liver transplant (LT) candidates [37]. Up to 80% of individuals with cirrhosis awaiting LT exhibit signs of malnutrition, sarcopenia, or micronutrient deficiencies [38]. Pre-transplant nutritional status is directly linked to waitlist mortality, susceptibility to infections, postoperative recovery, and both ICU and overall hospital length of stay.

All patients listed for liver transplantation should undergo comprehensive nutritional screening. Recommended tools include the Subjective Global Assessment (SGA) or the Royal Free Hospital Global Assessment (RFH-GA). Sarcopenia should be evaluated using cross-sectional imaging (e.g., CT-based assessment of the psoas or L3 skeletal muscle area). Dietary intake analysis and functional performance measures—such as handgrip strength and, where feasible, gait speed—are also essential. Notably, even individuals with obesity may present with sarcopenic obesity, which is associated with poorer post-transplant outcomes [39].

Computed tomography (CT)-based imaging is currently considered the gold standard for assessing sarcopenia in patients with liver disease, particularly in the pre-transplant or ICU setting. The most widely used method involves measuring skeletal muscle area (SMA) at the third lumbar vertebra (L3) level, using cross-sectional CT images. This can be carried out by quantifying either the total skeletal muscle area (TMA) at L3, normalized to height to calculate the skeletal muscle index (SMI), or measuring psoas muscle area or thickness as a surrogate for global muscle mass.

This method correlates strongly with outcomes such as mortality, complications, and transplant eligibility. However, limitations include radiation exposure, the need for recent imaging, and reliance on dedicated software (e.g., SliceOmatic 6, ImageJ 1.54p, or AI-based tools) for segmentation and analysis. These constraints limit real-time bedside applicability, particularly in resource-limited or emergency settings.

Emerging alternatives include ultrasound-based measurements, which offer a bedside, radiation-free option. Among these, the measurement of quadriceps muscle thickness, especially the rectus femoris cross-sectional area, has shown promising correlation with CT-defined sarcopenia and patient outcomes. Ultrasound is repeatable, inexpensive, and feasible in both ward and ICU environments, though standardization and operator training remain necessary for consistent use [40,41].

The primary goal of pre-transplant nutritional therapy is to optimize nutritional reserves and reduce modifiable risks prior to surgery. Energy intake should be targeted at 30–35 kcal/kg/day, with protein provision of 1.2–1.5 g/kg/day, and higher amounts in catabolic states. Frequent meals, including a late evening snack, are recommended to minimize overnight catabolism. If oral intake is inadequate, oral nutritional supplements (ONSs) should be used. In patients with refractory hepatic encephalopathy, branched-chain amino acid (BCAA)-enriched formulas may be beneficial.

### 9.1. Micronutrient Management

Micronutrient deficiencies are frequently observed in patients awaiting liver transplantation and should be systematically identified and managed. Several key micronutrients warrant particular attention due to their clinical relevance in this population. Zinc plays a crucial role in ammonia metabolism and immune function, and its deficiency may exacerbate hepatic encephalopathy. Thiamine is especially important in individuals with alcohol-related liver disease, where chronic alcohol use increases the risk of depletion. Vitamin D deficiency is also common and has been associated with an increased risk of post-transplant infections. In cases of cholestasis and impaired bile secretion, deficiencies in fat-soluble vitamins—namely vitamins A, E, and K—are frequently observed. Additionally, iron and folate levels should be assessed in the context of anemia, which is prevalent among patients with end-stage liver disease. Routine laboratory evaluations, alongside careful monitoring of dietary intake, are essential to guide individualized micronutrient supplementation strategies [42].

### 9.2. Nutrition Support in the ICU Before Transplantation

In critically ill patients awaiting liver transplantation, nutritional support is essential to optimize outcomes and minimize complications. Enteral nutrition (EN) is the preferred route if the gastrointestinal tract is functional, as it helps maintain mucosal integrity and modulate immune responses. Parenteral nutrition (PN) should be initiated when EN is contraindicated or fails to meet energy and protein requirements. Special attention must be given to the prevention and monitoring of refeeding syndrome, particularly in severely malnourished patients. Given the dynamic nature of critical illness, daily nutritional reassessment is imperative to ensure adequacy and appropriateness of therapy.

## 10. Refeeding Syndrome

Refeeding syndrome (RFS) is a critical consideration in the nutritional management of malnourished patients, particularly those with chronic liver failure (CLF) or acute-on-chronic liver failure (ACLF) in the intensive care unit (ICU) [43,44,45,46,47].

### 10.1. Definition and Pathophysiology

Refeeding syndrome is a potentially fatal condition resulting from the rapid reintroduction of nutrition in malnourished individuals. It is characterized by severe shifts in fluids and electrolytes, particularly phosphate, potassium, and magnesium, leading to metabolic and clinical complications. The pathophysiology involves insulin secretion in response to carbohydrate intake, which promotes cellular uptake of glucose and electrolytes, exacerbating existing deficiencies [48].

Patients at high risk for refeeding syndrome (RFS) include those with a history of prolonged fasting or minimal nutritional intake, typically exceeding five days. Additional risk factors encompass significant unintentional weight loss—defined as greater than 10% over the preceding three to six months—alongside a low body mass index (BMI < 18.5 kg/m^2^), chronic alcoholism, and uncontrolled diabetes mellitus. Electrolyte disturbances prior to refeeding, particularly hypophosphatemia, hypokalemia, and hypomagnesemia, further compound the risk. Certain medications, including insulin, chemotherapeutic agents, diuretics, and antacids, can exacerbate metabolic imbalances during refeeding. These risk factors are especially relevant in critically ill patients with chronic or acute-on-chronic liver failure (CLF or ACLF) who frequently present with multiple predisposing conditions concurrently, necessitating heightened vigilance and individualized refeeding strategies in the intensive care setting.

### 10.2. Clinical Manifestations

Symptoms of refeeding syndrome (RFS) can develop within 24 to 72 h after the initiation of nutritional support and may present with a range of potentially life-threatening complications. Hypophosphatemia is a hallmark feature and can result in muscle weakness, respiratory failure, and cardiac dysfunction. Hypokalemia may lead to arrhythmias and muscle cramps, while hypomagnesemia can manifest as tremors or seizures. Thiamine deficiency, often unmasked during refeeding, carries the risk of precipitating Wernicke’s encephalopathy. In addition, fluid overload is a common concern, with possible consequences including peripheral edema and congestive heart failure. These clinical manifestations highlight the critical need for close biochemical and clinical monitoring during the early phases of nutritional rehabilitation.

Implementing these strategies requires a multidisciplinary approach, involving physicians, dietitians, and nursing staff, to ensure safe and effective nutritional rehabilitation [49] (Table 8).

## 11. Refeeding Syndrome in ICU—Clinical Protocol

The following protocol represents a synthesis of evidence-based best practices, primarily derived from the National Institute for Health and Care Excellence (NICE) Clinical Guideline CG32 and the American Society for Parenteral and Enteral Nutrition (ASPEN) Consensus Recommendations on Refeeding Syndrome. It has been further adapted by the authors to reflect the specific challenges encountered in ICU patients with liver failure, including those with acute-on-chronic liver failure (ACLF) and sarcopenia. This protocol is designed to guide risk identification, biochemical monitoring, cautious nutritional advancement, and electrolyte correction in critically ill patients vulnerable to refeeding-related complications.

### 11.1. Identify Patients at Risk (Per NICE Guidelines)

Prior to initiating nutritional support, patients should be screened for risk factors associated with refeeding syndrome, particularly if any of the following criteria are met: a body mass index (BMI) below 16 kg/m^2^; unintentional weight loss exceeding 15% over a period of three to six months; minimal or no nutritional intake for more than five consecutive days; or a clinical history involving alcohol misuse or the use of medications such as diuretics, insulin, chemotherapy, or antacids. Additionally, the presence of electrolyte imbalances—including hypophosphatemia, hypokalemia, or hypomagnesemia—should prompt heightened caution. Patients at particularly high risk often include those with cirrhosis complicated by ascites and sarcopenia, individuals with acute-on-chronic liver failure (ACLF) characterized by systemic inflammation, and those with alcohol-related liver disease and severely compromised oral intake.

### 11.2. Baseline Lab Work

Before initiating nutritional therapy, it is essential to assess baseline biochemical parameters, including serum levels of phosphate, potassium, and magnesium, as these are most closely associated with the risk of refeeding syndrome. Additional measurements should include calcium, sodium, and glucose levels, as well as liver and kidney function tests. If available, thiamine levels should also be evaluated prior to refeeding. These laboratory parameters should be closely monitored on a daily basis for at least five to seven days following the initiation of nutritional support to promptly identify and manage emerging metabolic disturbances.

### 11.3. Nutritional Start Strategy

In patients identified as being at high risk for refeeding syndrome, nutritional support should be initiated cautiously. Energy intake should begin at approximately 10 to 15 kcal/kg/day and gradually be advanced to the full target over a period of 4 to 7 days. During the initial phase, the carbohydrate load should be limited to no more than 150 to 200 g/day to reduce the risk of metabolic complications. Protein intake should be maintained at 1.2 to 1.5 g/kg/day, provided that it is well tolerated. Rapid escalation of caloric intake should be avoided, and if clinically appropriate, enteral nutrition (EN) is the preferred route of administration due to its physiological benefits and lower risk of complications compared to parenteral nutrition.

### 11.4. Micronutrient Supplementation

Micronutrient supplementation should begin prior to the initiation of nutritional support and continue for 5 to 10 days to prevent or mitigate complications associated with refeeding. Thiamine is particularly critical and should be administered intravenously at a dose of 100 to 300 mg daily. A comprehensive multivitamin preparation and trace elements should also be provided, either intravenously or orally, depending on the clinical context. Zinc supplementation is recommended in cases of elevated ammonia levels or in patients with alcohol-related liver disease. Additional supplementation with folic acid, vitamin D, and fat-soluble vitamins (A, D, E, and K) should be considered based on individual deficiencies and clinical needs.

Clinical management of patients at risk for refeeding syndrome requires careful planning and monitoring (Table 9). Thiamine should be administered intravenously at a dose of 100 to 300 mg per day both prior to and during the refeeding process to prevent Wernicke’s encephalopathy. Serum electrolytes—particularly phosphate, potassium, and magnesium—should be monitored daily for at least 5 to 7 days following the initiation of nutrition. Special caution is warranted in patients with renal impairment or those on fluid-restricted regimens, as they may be more susceptible to volume overload or electrolyte shifts. Electrocardiographic (ECG) monitoring is recommended in cases of severe hypokalemia or hypomagnesemia, given the risk of life-threatening arrhythmias. Electrolyte abnormalities should be corrected gradually during ongoing nutritional support, with care taken to avoid overcorrection [50].

### 11.5. Fluid Management

Fluid management is a critical component of care during nutritional rehabilitation in high-risk patients. Sodium intake should be restricted to minimize the risk of fluid overload. Isotonic fluids should be administered with caution, particularly in individuals with compromised renal or cardiac function. Continuous monitoring is essential to detect early signs of fluid retention, including peripheral edema, pulmonary congestion, and cardiac strain.

### 11.6. Monitoring and Team Involvement

Electrolyte levels should be reassessed daily for a minimum of 5 to 7 days following the initiation of nutritional support. Based on clinical and biochemical responses, the nutrition plan should be reviewed and adjusted at least every 48 h to ensure safe and effective refeeding.

## 12. Postoperative Nutrition in Liver Transplant Recipients

Liver transplantation is associated with significant metabolic stress, characterized by a profound catabolic state that is further exacerbated by a systemic inflammatory response, immunosuppressive therapy, and an elevated risk of postoperative infections. During the immediate postoperative phase in the intensive care unit (ICU), patients frequently exhibit hypermetabolism and hypercatabolism, along with markedly increased protein turnover and substantial nitrogen losses. Additionally, immunosuppressive regimens contribute to various metabolic disturbances, including hyperglycemia and shifts in electrolyte balance. In this context, prompt and individualized nutritional therapy is critical to support graft function, facilitate immune recovery, and promote effective wound healing [38,51,52].

### Timing of Nutrition Support

Initiating early nutritional support within the first 24 h following liver transplantation (LT) is strongly recommended in the absence of contraindications. Early enteral feeding plays a critical role in preserving gut mucosal integrity and maintaining a stable microbiota, which in turn reduces bacterial translocation and modulates the systemic stress response. Enteral nutrition (EN) is generally safe and feasible in most patients within 12 to 24 h postoperatively, provided there is no hemodynamic instability, active gastrointestinal bleeding or ischemia, uncontrolled ileus, or a significant risk of aspiration.

Postoperative energy requirements typically range between 30 and 35 kcal/kg/day. Protein needs are elevated, often requiring 1.5 to 2.0 g/kg/day, to support tissue regeneration, immune competence, and overall recovery. These requirements may further increase in the presence of infections, reoperations, or severe sarcopenia. While indirect calorimetry remains the gold standard for determining energy expenditure, its availability in clinical settings is often limited, necessitating reliance on predictive equations and clinical judgment.

Nutrition should be reassessed daily in the ICU and then every 48–72 h during step-down care (Table 10).

## 13. Conclusions

Nutritional support is a critical, yet often underestimated, aspect of intensive care for patients with liver failure. Both acute and chronic forms of hepatic dysfunction are associated with profound metabolic disturbances, malnutrition, and catabolic stress, all of which negatively impact outcomes. Timely and individualized nutritional therapy—focused on adequate energy and protein provision, micronutrient repletion, and prevention of complications like refeeding syndrome—is essential for mitigating catabolism and supporting organ function, which may improve outcomes, reduce ICU length of stay, and enhance recovery.

Enteral nutrition should be prioritized whenever feasible, and clinical judgment is required to adjust caloric and protein goals based on disease severity, encephalopathy, and organ function. Tools such as the Subjective Global Assessment (SGA) and Royal Free Hospital Global Assessment (RFH-GA) allow for structured risk evaluation. In transplant candidates and recipients, perioperative nutritional care directly influences postoperative outcomes.

A multidisciplinary approach—engaging hepatologists, intensivists, dietitians, and pharmacists—is essential for optimal care. Future prospective studies must focus on validating nutritional interventions that target the unique inflammatory and metabolic phenotype of ACLF, and on developing practical, non-invasive bedside tools to quantify sarcopenia, allowing for more precise risk stratification and timely nutritional therapy.

## Figures and Tables

**Table 1:** King’s College Criteria for emergency liver transplantation in acute liver failure.

Etiology	Criteria
Acetaminophen-induced ALF	Arterial pH < 7.3 (any encephalopathy grade) OR INR > 6.5, creatinine > 3.4 mg/dL, grade III–IV HE
Non-acetaminophen ALF	INR > 6.5 OR 3 of: age < 10 or >40, jaundice > 7 days before HE, INR > 3.5, bilirubin > 17.5 mg/dL

Abbreviations: HE = hepatic encephalopathy; INR = international normalized ratio; and ALF = acute liver failure.

**Table 2:** Recommendations for nutritional therapy in the ICU for patients with acute liver failure.

Parameter	Recommendation for ICU Patients with ALF
Caloric intake	25–30 kcal/kg/day adjusted for stress and organ support
Protein intake	1.2–1.5 g/kg/day, even in hepatic encephalopathy (no restriction recommended)
Feeding route	Prefer enteral nutrition within 24–48 h if hemodynamically stable
Parenteral nutrition	Consider only if enteral feeding is contraindicated or insufficient
Feeding pattern	Continuous or cyclic feeding to minimize catabolism
Micronutrient support	Zinc, thiamine, selenium, phosphate, and ADEK vitamins
Refeeding monitoring	Watch phosphate, magnesium, and glucose, especially in malnourished patients

Abbreviations: BCAAs = branched-chain amino acids; EN = enteral nutrition; PN = parenteral nutrition; HE = hepatic encephalopathy; ICU = intensive care unit; and ALF = acute liver failure.

**Table 4:** Nutritional interventions in cirrhosis and supporting evidence.

Intervention	Clinical Benefits	Evidence/Reference
Late evening snack (LES)	Improves nitrogen balance, reduces catabolism, and lowers ammonia	[22,23]
BCAA supplementation	Reduces HE episodes, improves psychometric function, and preserves muscle	[19,20,21]
Plant-based protein	Better tolerated in HE, lower ammonia production, and higher fiber	[17,18]
Adequate protein intake	Prevents sarcopenia, supports immune function, and improves survival	[24]
Enteral nutrition (EN)	Preserves gut integrity, reduces infections, and improves outcomes	General physiological principles
Micronutrient supplementation	Zinc, thiamine, selenium, and ADEK support HE control and immunity	[17]

Abbreviations: LES = late evening snack; BCAAs = branched-chain amino acids; HE = hepatic encephalopathy; EN = enteral nutrition; and ADEK = vitamins A, D, E, and K.

**Table 5:** Nutritional targets and micronutrients in ACLF, adapted from EASL [35] and AASLD [11].

Parameter	Recommendation
Energy	25–30 kcal/kg/day in ACLF, adjusted for stress
Protein	1.2–1.5 g/kg/day; no restriction, even in HE
Feeding initiation	Start within 24–48 h if no contraindications
Feeding method	Enteral preferred; parenteral if enteral not feasible after 5–7 days
Micronutrients	Zinc, thiamine, selenium, and ADEK vitamins; phosphate and magnesium—especially during feeding
Diagnostic tools	Use CLIF-C ACLF or NACSELD-ACLF scores to assess organ failure and mortality risk

Abbreviations: ACLF = acute-on-chronic liver failure; HE = hepatic encephalopathy; ADEK = vitamins A, D, E, and K; CLIF-C = Chronic Liver Failure Consortium; and NACSELD = North American Consortium for the Study of End-Stage Liver Disease.

**Table 6:** Nutritional adjustments needed during CRRT in critically ill patients.

Aspect	Nutritional Implication
Protein	Increased catabolism and dialytic loss → 1.5–2.0 g/kg/day may be needed
Energy	Losses through dialysate modest → 25–30 kcal/kg/day remain appropriate
Electrolytes	Daily replacement of phosphate, magnesium, and potassium is essential
Water-soluble vitamins	Significant CRRT-associated losses of thiamine (200–300 mg/day IV), B6, C, folate → consider daily IV supplementation
Trace elements	Zinc and selenium may also be depleted; monitor and supplement as needed
Feeding route	Enteral nutrition is preferred if GI function is preserved; PN may be needed with fluid restriction or intolerance

Abbreviations: CRRT = continuous renal replacement therapy; PN = parenteral nutrition; and GI = gastrointestinal.

**Table 7:** Comparison of energy and protein targets between stable CLF and critically ill ACLF patients.

Parameter	CLF (Stable)	ACLF (Critically Ill)
Energy	30–35 kcal/kg/day	25–30 kcal/kg/day (adjusted for metabolic stress and tolerance)
Protein	1.2–1.5 g/kg/day	1.2–1.5 g/kg/day (including in HE; no restriction)
Meal pattern	Frequent meals + late evening snack (LES)	Continuous or intermittent enteral feeding
Feeding route	Oral preferred, enteral if needed	Enteral preferred; parenteral only if enteral is contraindicated

Abbreviations: CLF = chronic liver failure; ACLF = acute-on-chronic liver failure; HE = hepatic encephalopathy; and LES = late evening snack.

**Table 8:** Recommendations for prevention and management of refeeding syndrome in patients with liver disease.

Intervention Area	Recommendations
Assessment and monitoring	Identify high-risk patients (history of starvation, alcoholism, significant weight loss, and baseline electrolyte abnormalities). Monitor daily weight, fluid status, and electrolytes.
Nutritional strategy	Start feeding at 10–20 kcal/kg/day, progress gradually over 4–7 days; prioritize enteral nutrition if feasible.
Electrolyte replacement	Correct phosphate, potassium, and magnesium before and during refeeding. Supplement thiamine 200–300 mg/day.
Fluid management	Avoid fluid overload; monitor for signs of cardiac failure; adjust fluid and sodium intake accordingly.

**Table 9:** Electrolyte and micronutrient correction protocol prior to refeeding in patients with liver failure (adapted from ESPEN guidelines).

Element	Threshold for Correction	Recommended Replacement
Phosphate	<0.65 mmol/L	0.32–0.64 mmol/kg/day IV over 6 h
Potassium	<3.0 mmol/L	20–40 mmol IV over 4–6 h
Magnesium	<0.7 mmol/L	0.5 mmol/kg/day IV
Thiamine	—	100–300 mg IV daily for at least 3 days before refeeding

Abbreviations: IV = intravenous; ESPEN = European Society for Clinical Nutrition and Metabolism.

**Table 10:** Route of nutrition and related monitoring considerations in liver failure patients.

Aspect	Details/Recommendations
First choice	Enteral nutrition (nasogastric or post-pyloric)
Enteral details	- Start trophic feeds (10–20 mL/h), advance cautiously - Monitor residuals, diarrhea, and ileus
Parenteral nutrition	- Use if EN contraindicated or poorly tolerated - Lipids: use cautiously, monitor triglycerides - Glucose: avoid overload, especially with steroid-induced hyperglycemia
Micronutrient support	- Zinc and selenium for antioxidant/graft function - Thiamine, vitamin D, and B12 for recovery - Monitor hypophosphatemia and magnesium depletion - Close glucose monitoring (tacrolimus/steroids)
Monitoring and adjustment	- Daily energy intake vs. target - GI tolerance (GRVs, diarrhea, and bowel sounds) - Signs of infection or graft dysfunction - Adjust for renal/pulmonary complications

Abbreviations: EN = enteral nutrition; PN = parenteral nutrition; and GRV = gastric residual volume.

## Data Availability

Not applicable.

## Abbreviations

The following abbreviations are used in this manuscript:

AAAs	Aromatic amino acids
ACLF	Acute-on-chronic liver failure
ADEK	Vitamins A, D, E, and K
ALF	Acute liver failure
BCAAs	Branched-chain amino acids
BMI	Body mass index
CLF	Chronic liver failure
CRRT	Continuous renal replacement therapy
EN	Enteral nutrition
GRV	Gastric residual volume
HE	Hepatic encephalopathy
HRS	Hepatorenal syndrome
IC	Indirect calorimetry
ICU	Intensive care unit
INR	International normalized ratio
LES	Late evening snack
LT	Liver transplantation
MELD	Model for End-Stage Liver Disease
OLT	Orthotopic liver transplantation
PN	Parenteral nutrition
REE	Resting energy expenditure
RFH-GA	Royal Free Hospital Global Assessment
RRT	Renal replacement therapy
SBP	Spontaneous bacterial peritonitis
SGA	Subjective Global Assessment
